# Glycogen storage diseases: Twenty‐seven new variants in a cohort of 125 patients

**DOI:** 10.1002/mgg3.877

**Published:** 2019-09-11

**Authors:** Fernanda Sperb-Ludwig, Franciele Cabral Pinheiro, Malu Bettio Soares, Tatiele Nalin, Erlane Marques Ribeiro, Carlos Eduardo Steiner, Eugênia Ribeiro Valadares, Gilda Porta, Carolina Fishinger Moura de Souza, Ida Vanessa Doederlein Schwartz

**Affiliations:** ^1^ Post‐Graduation Program in Genetics and Molecular Biology Universidade Federal do Rio Grande do Sul Porto Alegre Brazil; ^2^ Laboratory of Basic Research and Advanced Investigations in Neurosciences (BRAIN) Hospital de Clínicas de Porto Alegre Porto Alegre Brazil; ^3^ Hospital Infantil Albert Sabin Fortaleza Brazil; ^4^ Universidade Estadual de Campinas Campinas Brazil; ^5^ Departamento de Propedêutica Complementar Faculdade de Medicina da Universidade Federal de Minas Gerais Belo Horizonte Brazil; ^6^ Hospital Infantil Menino Jesus São Paulo Brazil; ^7^ Medical Genetics Service Hospital de Clínicas de Porto Alegre Porto Alegre Brazil

**Keywords:** glycogen storage disease, hepatic GSD, molecular diagnosis, next‐generation sequencing

## Abstract

**Background:**

Hepatic glycogen storage diseases (GSDs) are a group of rare genetic disorders in which glycogen cannot be metabolized to glucose in the liver because of enzyme deficiencies along the glycogenolytic pathway. GSDs are well‐recognized diseases that can occur without the full spectrum, and with overlapping in symptoms.

**Methods:**

We analyzed a cohort of 125 patients with suspected hepatic GSD through a next‐generation sequencing (NGS) gene panel in Ion Torrent platform. New variants were analyzed by pathogenicity prediction tools.

**Results:**

Twenty‐seven new variants predicted as pathogenic were found between 63 variants identified. The most frequent GSD was type Ia (*n* = 53), followed by Ib (*n* = 23). The most frequent variants were p.Arg83Cys (39 alleles) and p.Gln347* (14 alleles) in *G6PC* gene, and p.Leu348Valfs (21 alleles) in *SLC37A4* gene.

**Conclusions:**

The study presents the largest cohort ever analyzed in Brazilian patients with hepatic glycogenosis. We determined the clinical utility of NGS for diagnosis. The molecular diagnosis of hepatic GSDs enables the characterization of diseases with similar clinical symptoms, avoiding hepatic biopsy and having faster results.

## Abstract 2

The study presents the largest cohort ever analyzed in Brazilian patients with hepatic glycogenosis. Thirty new mutations predicted as pathogenic were found in the 65 variants identified. We determined the clinical utility of next‐generation sequence (NGS) for diagnosis. The molecular diagnosis of hepatic GSDs enables the characterization of diseases with similar clinical symptoms, avoiding hepatic biopsy and having faster results.

## 1 INTRODUCTION

Hepatic glycogen storage diseases (GSDs) are a group of inborn errors of metabolism that include 11 different diseases caused by defects in glycogenolytic pathway. These defects are caused by pathogenic variants that result in enzymatic deficiencies for glycogen breakdown or synthesis, or problems in proteins that regulate glycogen metabolism. The consequence is accumulation of glycogen in tissues, especially in liver (Chen & Zhong, 2013).

The general GSD frequency is 1 in 2,000–43,000 and their distribution is pan‐ethnic (Özen, 2007; Vega et al., 2016). Some forms of GSDs are underestimated due to mild symptoms, rare occurrence, or difficult diagnostic methods. Symptoms may range from neonatal to almost asymptomatic, and the age of onset, severity, morbidity, mortality, and prognosis are dependent of causal variants (Kishnani et al., 2014, 2010; Laforêt, Weinstein, & Smit, 2012; Özen, 2007; Wang et al., 2012). The main clinical symptoms are hypoglycemia and hepatomegaly, and long‐term complications are frequent (Burda & Hochuli, 2015).

Different types of GSDs can be clinically indistinguishable and need liver biopsy, an invasive method. In this aim, the molecular diagnosis using blood samples generates an accurate diagnosis and allows prognosis and genetic counseling (Choi et al., 2017; Davit‐Spraul et al., 2011). Similar diseases in clinical symptoms, metabolic routes, or genetic features are a challenge to diagnose. In this aim, next‐generation sequencing (NGS) is an important tool to determine the cause of the disease with accuracy and efficacy, allowing a more suitable treatment.

Only two previous studies have characterized 13 patients with GSD Ia and Ib in Brazilian population (Carlin, Scherrer, Tommaso, Bertuzzo, & Steiner, 2013; Reis et al., 2001).

In the present study, we describe the results of variant analysis in a cohort of 125 patients with hepatic GSD suspected diagnosis by NGS.

## 2 MATERIAL AND METHODS

This study was approved by the Research Ethics Committee of Hospital de Clínicas de Porto Alegre (project no. 15‐0556), and all patients and guardians provided written informed consent for participation.

Were analyzed 125 patients with clinical symptoms of hepatic GSD. Blood samples were collected in EDTA vacuum container. DNA was extracted with Easy‐DNA Purification kit (Thermo Fisher). DNA samples were quantificated in NanoDrop 1000 (Thermo Fisher) and through Qubit dsDNA HS Assay Kit (Thermo Fisher).

The gene panel amplicon was designed with Ion Ampliseq Designer software (Thermo Fisher), and included the exons and flanking 40 bp into introns of 11 genes involved in hepatic GSD (Table 1). The sequencing was performed in Ion Torrent PGM platform (Applied Biosystems), based in PCR amplification with minimal coverage of 200X. Base calling and sequence read quality assessments were performed using Torrent Suite 5.0.5. Alignment of the sequence reads to a reference human genome (GRCh37.p13) was performed using IonStates alignment.

**Table 1:** Genes and diseases diagnosed in gene panel

GSD type	Gene	OMIM	Location	Enzyme deficiency	Inheritance	Incidence	Main clinical symptoms			Mutations in HGMD
							Hypoglycemia	Hepatomegaly	Hyperlipidemia	
0	GYS2	138571	*12p12.2*	glycogen synthase	AR		Yes	No	No	18 (19)
Ia	*G6PC*	232200	*17q21*	Glucose‐6‐phosphatase	AR	1 in 50,000–100,000	Yes	Yes	Yes	106 (111)
Ib	*SLC37A4*	232220	*11q23.3*	Glucose‐6‐phosphate transporter	AR		Yes	Yes	Yes	101 (110)
III	AGL	232400	*1p21*	glycogen debranching enzyme	AR	1 in 100,000	Yes	Yes	Yes	155 (239)
IV	*GBE1*	232500	*3p12.3*	glycogen branching enzyme	AR	1 to 500,000	No	Yes	No	50 (69)
VI	*PYGL*	232700	*14q21‐q22*	liver glycogen phosphorylase	AR	1 in 65,000–85,000	Yes	Yes	Yes	31 (43)
XIa	*PHKA2*	306000	*Xp22.2‐p22.1*	phosphorylase kinase α subunit	X‐linked		Yes	Yes	No	80 (104)
XIb	*PHKB*	261750	*16q12‐q13*	phosphorylase kinase β subunit	AR		Yes	Yes	No	18 (24)
XIc	*PHKG2*	613027	*16p12.1‐p11.2*	phosphorylase kinase γ subunit	AR		Yes	Yes	Yes	19 (31)
XI	*SLC2A2*	612933	*3q26.1‐q26.2*	facilitated glucose transporter	AR		Yes	Yes	Yes	66 (78)
XII	*ALDOA*	611881	*16q22‐q24*	aldolase A	AR		No	Yes	No	7 (8)

The softwares Enlis Genome Research (LLC), Variant Effect Predictor (Ensembl), Ion Reporter (Thermo Fisher) and Varstation^®^ (Varstation) were used to detect and classify variants. To determine the variants causing disease, the following were considered: ACMG guideline (Richards et al., 2015); allele frequency under 1% in the 1,000 Genomes Project (Sabeti, 2015); location in exon or borderlines; impact in the protein (missense, nonsense or splicing sites); and pathogenicity by predictors SIFT and Polyphen 2. For score of pathogenicity predictions in missense novel variants were used the softwares Polyphen 2 (Adzhubei et al., 2010), SIFT (Vaser, Adusumalli, Leng, Sikic, & Ng, 2016), PROVEAN (Choi, Sims, Murphy, Miller, & Chan, 2012), Mutation Teaster (Schwarz, Cooper, Schuelke, & Seelow, 2014), Pmut 2017 (López‐Ferrando, Gazzo, Cruz, Orozco, & Gelpí, 2017), SNP&Go (Profiti, Martelli, & Casadio, 2017), PhDSNP (Capriotti & Fariselli, 2017), Panther (Thomas et al., 2003), SNAP2 (Hecht, Bromberg, & Rost, 2015) and MutPred (Pejaver et al., 2017). For splice site variants, Genescan (Burge & Karlin, 1997) and MaxEntScan were used (Yeo & Burge, 2003).

Validations of NGS results were realized by Sanger sequencing in patients and in parents when the sample was available. The unbiased capture and deep coverage of each coding exon and adjacent intronic region of all genes in this panel ensure accuracy of variant detection.

## 3 RESULTS

We analyzed 125 patients with clinical suspicion of hepatic GSD. All samples were successfully sequenced. We found 63 different variants in 110 families, and 27 of those were new variants (Tables 2 and Appendix S1).

**Table 2:** Variants found in 125 patients with hepatic GSDs, their references, frequencies in databanks, and ACMG classification

Gene GenBank	Allele	Protein	Location	Reference	ExAC Frequence	ACMG	
*G6PC NM_000151*	c.70C > T	p.Gln24*	E1	Rocha, Cabral, and Vilarinho (2000)		PM2, PVS1	Probably pathogenic
	c.77delC	p.Ser26fs	E1	Lei et al. (1995)^#^	0.00006	PM2, PP5, PVS1	Pathogenic
	c.113A > T	p.Asp38Val	E1	ChevalierPorst et al. (1996)	0.000008	PM1, PM2, PP2, PP3, PP5	Probably pathogenic
	c.161A > C	p.Gln54Pro	E1	Trioche et al. (2000)		PM1, PM2, PP2, PP3	Probably pathogenic
	c.189G > C	p.Trp63Cys	E1	New		PM1, PM2, PP2, PP3	Probably pathogenic
	c.202G > A	p.Gly68Arg	E1	Reis et al. (2001)		PM1, PM2, PP2, PP3	Probably pathogenic
	c.231−1G > A		I1	Akanuma et al. (2000)		PM2	Uncertain
	c.247C > T	p.Arg83Cys	E2	Lei et al. (1993)	0.0005	BS1, PM1, PP2, PP3, PP5	Uncertain
	c.323C > T	p.Thr108Ile	E2	Trioche et al. (2000)		PM1, PM2, PP2, PP3	Probably pathogenic
	c.401_402delCT	p.Thr134 = fs	E3	New		PM2	Uncertain
	c.439A > T	p.Arg147*	E3	New*	0.000008	PM1, PM2, PVS1	Pathogenic
	c.509G > A	p.Arg170Gln	E4	Barkaoui et al. (2007)	0.00001	PM1, PM2, PP2, PP3	Probably pathogenic
	c.563‐3C > G		I4	Kishnani et al. (2009)		PM2	Uncertain
	c.809G > T	p.Gly270Val	E5	Lei et al. (1995)	0.00001	PM2, PP2, PP3	Uncertain
	c.969C > A	p.Tyr323*	E5	Calderaro et al. (2013)	0.000008	PM2, PVS1	Probably pathogenic
	c.1012G > T	p.Val338Phe	E5	Rake et al. (2000)	0.00001	PM2, PP2, PP3	Uncertain
	c.1039C > T	p.Gln347*	E5	Lei, Pan, Shelly, Liu, and Chou (1994)	0.0002	BS1, PP5, PVS1	Uncertain
*SLC37A4 NM_001467*	c.59G > A	p.Gly20Asp	E3	Veiga da Cunha et al. (1998)	0.00001	PM1, PM2, PP2, PP3	Probably pathogenic
	c.92_94delTCT	p.Phe31_Ser32del	E3	New		PM2	Uncertain
	c.344_345dupGG	p.Leu116Glyfs	E4	Galli et al. (1999)	0.00001	PM2, PVS1	Probably pathogenic
	c.446G > A	p.Gly149Glu	E5	Galli et al. (1999)	0.00002	PM1, PM2, PP2, PP3	Probably pathogenic
	c.547T > C	p.Cys183Arg	E5	Veiga da Cunha et al. (1998)		PM1, PM2, PP2, PP3	Probably pathogenic
	c.557T > C	p.Leu186Pro	E5	New		PM1, PM2, PP2, PP3	Probably pathogenic
	c.703_705delGTG	p.Val236del	E7	Hou et al. (1999)		PM2, PP5	Uncertain
	c.899G > A	p.Arg300His	E9	Marcolongo et al. (1998)	0.00003	PM1, PM2, PP2, PP3	Probably pathogenic
	c.1042_1043delCT	p.Leu348fs	E9	Veiga da Cunha et al. (1998)		BS1, PP5, PVS1	Uncertain
	c.1179G > A	p.Trp393*	E10	New*	0.00002	PM2, PVS1	Probably pathogenic
*AGL NM_000642*	c.293 + 2T>A		I3	Hadjigeorgiou et al. (1999)		PM2	Uncertain
	c.325G > T	p.Val109Leu	E4	New*	0.00009	BP1, PM1, PM2, PP3,	Uncertain
	c.744G > A	p.Trp248*	E6	New		PM1, PM2, PVS1	Pathogenic
	c.1383G > A	p.Trp461*	E11	New*	0.000008	PM1, PM2, PVS1	Pathogenic
	c.1481G > A	p.Arg494His	E12	Goldstein et al. (2010)	0.008	BP1, BS1, PM1, PP3	Probably Benign
	c.1571G > A	p.Arg524His	E12	Lucchiari et al. (2006)	0.000008	BP1, PM1, PM2, PP3	Uncertain
	c.1734A > T	p.Arg578Ser	E13	New		BP1, PM1, PM2, PP3	Uncertain
	c.1858_1859delCT	p.Leu620Valfs	E14	New		PM2, PVS1	Probably pathogenic
	c.2455_2458delAAAC	p.Lys819 = fs	E19	New		PM2, PVS1	Probably pathogenic
	c.2728C > T	p.Arg910*	E21	Lucchiari et al. (2006)	0.000008	PM1, PM2, PVS1	Pathogenic
	c.2904_2905insT	p.Tyr969Leufs	E22	New		PM2, PVS1	Probably pathogenic
	c.3214_3215delGA	p.Glu1072Aspfs	E24	Goldstein et al. (2010)	0.000008	PM2, PVS1	Probably pathogenic
	c.3475_3476insA	p.Gln1159fs	E26	New		PM2, PVS1	Probably pathogenic
	c.3484C > T	p.Gln1162*	E26	New		PM1, PM2, PVS1	Pathogenic
	c.3625C > T	p.Gln1209*	E27	New		PM1, PM2, PVS1	Pathogenic
	c.3814_3815delG	p.Arg1272 = fs	E28	New		PM2, PVS1	Probably pathogenic
	c.3904delA	p.Lys1302fs	E29	New		PM2, PVS1	Probably pathogenic
	c.3980G > A	p.Trp1327*	E30	Lucchiari et al. (2002)	0.00002	PM1, PM2, PVS1	Pathogenic
	c.4528_4529insA	p.Tyr1510*	E34	Shen and Chen (2002)	0.00001	PM2, PVS1	Probably pathogenic
*PYGL NM_001163940*	c.131G > A	p.Arg44His	E1	Hoogeveen et al. (2015)		PM2, PP2, PP3	Uncertain
	c.697G > A	p.Gly233Ser	E6	New*	0.00004	PM2, PP2, PP3	Uncertain
*PHKA2 NM_000292*	c.133C > T	p.Arg45Trp	E2	Beauchamp et al. (2007)		PM1, PM2, PP2, PP3	Probably pathogenic
	c.557G > A	p.Arg186His	E6	Burwinkel et al. (1996)		PM1, PM2, PP2, PP3, PP5	Probably pathogenic
	c.883C > T	p.Arg295Cys	E9	Ban, Sugiyama, Goto, Mizutani, and Togari (2003)		PM1, PM2, PP2, PP3, PP5	Probably pathogenic
	c.1963 + 1G>A		I18	Rodríguez‐Jimenez et al. (2017)	0.00001	PM2	Uncertain
	c.2452C > T	p.Gln818*	E22	New		PM2, PVS1	Probably pathogenic
	c.3614C > T	p.Pro1205Leu	E33	van den Berg et al. (1995)		PM2, PP2, PP3, PP5	Uncertain
	c.3629G > A	p.Gly1210Glu	E33	Rudolfova, Slováčková, Trbušek, Pešková, and Kozák (2001)		PM2, PP2, PP3	Uncertain
*PHKB NM_000293*	c.572_576delAGATT	p.Gln191Hfs	E6	New*	0.000008	BS1, PVS1	Uncertain
	c.1972‐2A > G		I20	New		PM2	Uncertain
	c.2081C > G	p.Ser694*	E22	New		PM2, PVS1	Probably pathogenic
	c.2181delT	p.Leu728fs	E22	New		PM2, PVS1	Probably pathogenic
*PHKG2 NM_000294*	c.454C > T	p.Arg152*	E6	New*	0.000008	PM1, PM2, PVS1	Pathogenic
	c.502C > T	p.Arg168*	E6	Davit Spraul et al. (2011)	0.00001	PM1, PM2, PVS1	Pathogenic
	c.835C > T	p.Arg279Cys	E9	New		PM1, PM2, PP2, PP3	Probably pathogenic
	c.927 + 1G>A		I9	New		PM2	Uncertain

Note

E: Exon; I: Intron; New*: New mutation related to hepatic GSDs, however presented in data banks; #discordance between literature nomenclature and HGVS rules.

Seventy‐five patients are men. The patients included in the study are from all Brazilian regions: 63 from the southeast (SP *n* = 48, RJ *n* = 2, MG *n* = 10, ES *n* = 3), 50 from the south (RS *n* = 46, SC *n* = 4), eight from the northeast (BA *n* = 2, CE *n* = 3, PB *n* = 3), two from the Midwest (DF = 1, MT *n* = 1) and two from the north (PA *n* = 2) (Appendix S1).

Both pathogenic variants were identified in 118 patients confirming the molecular diagnosis of hepatic GSD. For two patients, only one variant was found (patients 84 and 85). In five patients, no variant was identified (Appendix S1). All identified variants were confirmed by Sanger sequencing and investigated in literature or databanks (Tables 2 and Appendix S1).

Eight families included in this study had multiple affected individuals. For patients 5, 11, and 12 (11 and 12 are sisters), their parents reported consanguinity (Appendix S1). These information were considered while counting alleles.

Sixty‐three alleles were identified, in which 26 are missense variants (41.2%), 16 are nonsense variants (25.3%), six are splice site variants (9.5%), 11 deletions (17.4%), three insertions (4.7%) and one duplication (1.5%) (Table 2).

Among the 125 patients analyzed, 53 were genetically diagnosed with GSD Ia (42%), 23 with GSD Ib (18%), 14 with GSD III (11%), two with GSD VI (1.6%), 16 GSD IXa (12%), six with GSD IXb (4.8%), six with GSD IXc (4.8%), and five were not diagnosed (4%) (Appendix S1).

The most frequent variants in patients were p.Arg83Cys, observed in 39 alleles (18.5%), and p.Gln347* present in 14 alleles (6.6%), both in *G6PC* gene, causing GSD Ia. The other frequent variant, p.Leu348Valfs in *SLC37A4* gene, was observed in 10% of alleles causing GSD Ib.

Variants not described in the literature were evaluated for protein impact by nine in silico pathogenicity prediction algorithms. All new missense variants were predicted as pathogenic. In the bioinformatics analyses of new splice site variants, all were confirmed to modify the exon–intron structures in different forms importantly, showing sufficient entropy forces to perform the incorrect splicing.

## 4 DISCUSSION

This is one of the largest screening of variants causing the different forms of GSDs in patients, including 125 patients and describing 63 different variants, of which 27 are novel.

The GSD Ia and GSD Ib represent 60% of the analyzed patients. In other analyzed cohorts, Vega et al. (2016) reported more than three‐quarters of patients who had GSD III or GSD IXa (39% of each type), and Özen (2007) found GSD type IXa as the most common form of the disease. These data reflect the differences among populations, the existence of private pathogenic variants, and the differences in prevalence of variants in GSDs.

GSD type Ia is the most widely distributed. The most frequent pathogenic variant found in this work was p.Arg83Cys, present in 18.5% of all patients and 39% of alleles in GSD Ia patients. This is one of the most important variant found around the world in patients with GSD Ia (Chou & Mansfield, 2008; Matern, Seydewitz, Bali, Lang, & Chen, 2002). This variant in *G6PC* is in the active center of the enzyme G6Pase and presented no detectable activity in transient expression assays (Lei, Shelly, Pan, Sidbury, & Chou, 1993). p.Arg83Cys is present in 50% of alleles in French and Tunisian patients (Barkaoui et al., 2007; Trioche et al., 2000), 80% of Sicilian and 100% of alleles in Ashkenazi Jewish patients (Ekstein et al., 2004; Stroppiano et al., 1999). This variant is found in genomAD in a frequency of 0.0005 (Lek et al., 2016) and appears to be in a hotspot since two other variants are observed in the same position (p.Arg83His and p.Arg83=). There are another eight variants in amino acids 80, 81, and 82, six of them being pathogenic. In the same gene, the variant p.Gln347* was in 6.9% of all alleles or in 15% of alleles in GSD Ia. They both represent approximately 54% of variants found in GSD Ia patients.

The second most frequently found variant among all patients was p.Leu348Valfs in *SLC37A4* gene, present in 10% of patients, and 47.7% of alleles (21/44 alleles) in GSD Ib. This variant was present in 39% of Serbians patients (Skakic et al., 2018) and 31% of White patients reviewed in Chou, Jun, and Mansfield (2010).

Twenty‐seven novel variants were identified among the 125 patients, observed mainly among patients with GSD type III and type IX. *AGL*, that causes GSD III, is one of the largest genes, and has the highest number of variants reported in HGMD – The Human Gene Mutation Database (Stenson et al., 2003), which proves its heterogeneity. The increased number of variants in type IX patients can be justified by their lower characterization.

Some of the novel variants have already been detected in database projects involving the search for variants in a large number of individuals but never related to patients. We investigate the variants in “The Exome Aggregation Consortium” – ExAC – composed of 60,706 unrelated individuals, and the Online Archive of Brazilian Mutations – AbraOM – composed of 609 elderly individuals, as in other databases (Lek et al., 2016; Naslavsky et al., 2017; Sabeti, 2015). Seven of 27 novel variants were in ExAC, all in very low frequencies (Table 2).

Seven different types of GSDs were found. Only types 0, IV, XI and XII were not observed among the 125 patients. The type IX represented 22.4% of the patients (12.8% of type IXa), since GSD type IX had never been described in Brazilian patients.

In two patients, only one variant was found (patients 84 and 85) instead of two. Both patients presented variants in *AGL*. This gene has the highest number of gaps in coverage of NGS and is one of the biggest genes in panel, with 36 exons. However, the gaps were analyzed by Sanger sequencing and no variant was found. The results obtained from these patients are contradictory, since the variants found in both cases are described for GSD type III; however both patients presented inconsistent clinical findings. One of them has Down syndrome and liver histology similar to GSD III, but no biochemical results are compatible with the disease and the patient does not present any clinical symptoms. The other patient presented hypoglycemia from birth, however, currently associated with hyponatremia and metabolic acidosis. The liver biopsy was inconclusive and not suggestive of GSD. Therefore, it is possible that variants or technical artifacts are eliminating the amplification of the mutated allele or the variants are in regulatory regions, not covered by the panel, but the absence of disease is a possibility (Hedell, Dufva, Ansell, Mostad, & Hedman, 2015; Inokuchi et al., 2016).

In five patients with no identified variants, the clinical suspicions are mild or inconclusive, once they did not have clear clinical indications or laboratory findings supporting the diagnosis of GSD, besides hypoglycemia and/or hepatomegaly. The NGS was a diagnostic exclusion test; therefore, it was an expected result. These patients probably do not have GSD, once hepatomegaly and hypoglycemia are difficult to distinguish from other metabolic storage disorders without more clinical findings. Another possibility is the presence of variants in nontargeted deep intronic and regulatory regions (Wang et al., 2013).

Relationships of synergistic heterogeneity should be considered for GSDs, since the disease‐causing deficient enzymes share metabolic pathways, however it was not observed in the present study (Vockley, Rinaldo, Bennett, Matern, & Vladutiu, 2000).

The patient 120 is possibly a GSD patient because he presented clinical symptoms such as hypoglycemia and ketonuria but no other clinical signs, however only synonymous variants were found in *GYS2* (that causes GSD type 0), which does not justify the disease.

This variety of results reflects the profile of an extremely large country with an interesting and important mix of people from all over the world. The presence of immigrants from the most diverse origins, such as Africans, Asians, and Amerindians justifies the variability of alleles found in a highly mixed population. Genetic analyses indicate that Latin Americans trace their ancestry mainly in the intermixing of Native Americans, Europeans, and Sub‐Saharan Africans. Historically, Latin America has a continuous, differential, and diverse intra‐ and intercontinental migration events, and presents higher prevalence of metabolic diseases (Adhikari, Chacón‐Duque, Mendoza‐Revilla, Fuentes‐Guajardo, & Ruiz‐Linares, 2017; Chacón‐Duque et al., 2018; Giolo et al., 2012; Quinto‐Sánches et al., 2017; Resque et al., 2016).

Among the advantages of NGS diagnosis, patients undiagnosed by traditional means were investigated and correctly diagnosed in the present study. This method is especially promising for mixed populations with high level of heterogeneity. This method also allows the identification of unexpected diagnoses in the supposed typical phenotypes. Rare genetic diseases can be a diagnostic challenge, sometimes an odyssey. The NGS technologies can provide a fast diagnosis, advantages for treatment management, in reproductive choices, genetic counseling, and fertility services (Schofield et al., 2017). The GSD traditional diagnosis methods involve liver biopsy, an invasive and risked method, that can be avoided with a well‐established molecular method (Bali, Chen, Austin, & Goldstein, 2016; Lévesque et al., 2016).

This diagnosis is an important advancement for patients with nontypical forms of disease, especially for those who need agile actions, since it evaluates 11 genes at the same time.

This was an important step to increase the knowledge about the genetics of the different types of hepatic GSDs in Brazilian patients, since they have a genetically heterogeneous origin, and it is reflected in the variability of types and variants (Vega et al., 2016; Wang et al., 2013). The evaluation by NGS also allows to detect cases of synergic heterogeneity that cannot be perceived by Sanger sequencing.

Differentiated therapeutic management among GSD justifies the population characterization of patients. If NGS analyses are not available or expensive, the molecular diagnosis should be conducted first through the search for the pathogenic variants p.Arg83Cys and p.Gln347* in *G6PC* in case of GSD Ia or p.Leu348Valfs in *SLC37A4* for GSD Ib. Sanger sequencing approach is the most cost‐effective to solve up to 40% of the cases. However, for the cases without prevalent mutations or without suspected type of GSD, NGS is the most effective solution.

This study emphasizes that molecular genetic analysis is a reliable and convenient alternative to the assay of enzymatic activity in a fresh liver biopsy specimen for the diagnosis of GSDs. This type of study is an important tool for the estimation of disease progression, since different types of GSDs present variations in their clinical course and treatment, besides serving as a basis for genetic counseling and prenatal diagnosis.

The discovery of a significant number of new mutations reinforces the allelic variability of different GSDs and proves that the diagnosis of GSDs in Brazil can be challenging, showing the validity of NGS gene panel use for diagnosis.

## CONFLICT OF INTEREST

The authors declare no conflict of interest.
